# Genome Sequence of *Bradyrhizobium pachyrhizi* Strain PAC48^T^, a Nitrogen-Fixing Symbiont of *Pachyrhizus erosus* (L.) Urb.

**DOI:** 10.1128/genomeA.01074-15

**Published:** 2015-09-17

**Authors:** Jakeline Renata Marçon Delamuta, Renan Augusto Ribeiro, Douglas Fabiano Gomes, Renata Carolina Souza, Ligia Maria Oliveira Chueire, Mariangela Hungria

**Affiliations:** a Embrapa Soja, Soil Biotechnology, CP 231, Londrina, Paraná, Brazil; b Capes, SBN, Lote 06, Edifício Capes, Brasília, Brazil; c CNPq, SHIS QI 1 Conjunto B, Lago Sul, Brasília, Brazil; d UFPR, Department of Microbiology, CP 19031, Curitiba, Paraná, Brazil

## Abstract

*Bradyrhizobium pachyrhizi* PAC48^T^ has been isolated from a jicama nodule in Costa Rica. The draft genome indicates high similarity with that of *Bradyrhizobium elkanii*. Several coding sequences (CDSs) of the stress response might help in survival in the tropics. PAC48^T^ carries *nodD1* and *nodK*, similar to *Bradyrhizobium* (*Parasponia*) ANU 289 and a particular *nodD2* gene.

## GENOME ANNOUNCEMENT

Legumes have broadly been used as food crops, forages, and green manure, especially due to their ability to establish symbiotic associations with nitrogen-fixing bacteria, giving a relevant contribution to the global N balance (1, 2). *Bradyrhizobium* is abundant in the tropics, where most soils are depleted of nitrogen, giving important contributions when associated with a variety of indigenous legumes (3, 4). The genus *Pachyrhizus* encompasses tuberous root-producing legume species known as yam bean, found particularly in Central America and used as sources of starch, oil, and protein (5, 6).

Taxonomic studies point to high genetic diversity among *Bradyrhizobium* strains (3–6), and based on the analysis of the 16S rRNA gene, the genus can be split in two great groups, the *Bradyrhizobium japonicum* and *Bradyrhizobium elkanii* superclades (3, 4, 6–8). The species *Bradyrhizobium pachyrhizi* was described as being related to the *B. elkanii* superclade (6). Here, we report the draft genome of *B. pachyrhizi* type strain PAC48^T^ (=LMG 24246^T^ =CECT 7396^T^ =CNPSo 2077^T^) isolated from a *Pachyrhizus erosus* nodule in Guanacaste, Costa Rica.

To access the bacterial genome sequence, total DNA was extracted using the DNeasy blood and tissue kit (Qiagen) and processed on the MiSeq platform (Illumina) at Embrapa Soja, Londrina, Brazil. Shotgun sequencing generated 6,109,424 paired-end reads (2 × 150 bp), corresponding to approximately 100-fold coverage. The FASTQ files were *de novo* assembled by Velvet (9). The genome analyses revealed that strain PAC48^T^ has one circular chromosome with high similarity to that of *B. elkanii*. Sequences were submitted to RAST (10) and the genome estimated to be 8,746,824 bp, assembled in 496 contigs. Annotation identified 8,213 CDSs, and 39% were classified in 498 subsystems. The major categories were of amino acids and derivatives, carbohydrates, cofactors, vitamins, prosthetic groups, and pigments.

*B. pachyrhizi* PAC48^T^ carries genes coding for type I, II, II/IV, III, IV, and V secretion systems; 89 CDSs were related to cell motility and chemotaxis, and 204 CDSs fit into the stress response category, half related to oxidative stress, which might be implicated in the tolerance of tropical-stressing conditions. In general, the organization of nodulation genes resembled that of *Bradyrhizobium diazoefficiens* USDA 110^T^ (11) (previously classified as *B. japonicum* [8]). However, *nodD1* and *nodK* showed higher similarity with *Bradyrhizobium* (*Parasponia*) strain ANU 289 and with the Brazilian strains SEMIAs 6160 and 6152, previously identified as forming a distinct subcluster within the *B. elkanii* superclade (12). In addition, PAC48^T^ possesses a *nodD2* gene showing similarity (NCBI database) to *B. elkanii* USDA 94 (89%) and with one uncultured rhizobium (74%). Recent studies have pointed out the importance of the regulatory *nodD* gene in determining host specificity and stress tolerance (13), one hypothesis that might also apply to *B. pachyrhizi*.

### Nucleotide sequence accession number.

The whole-genome shotgun project has been deposited at DDBJ/EMBL/GenBank under the accession number LFIQ00000000. The BioProject number is PRJNA287433, and the BioSample number is SAMN03782120. The version described in this paper is the first version.
